# Patient-Reported Quality of Life in Lung Transplantation: Prospective Cohort Study

**DOI:** 10.2196/86211

**Published:** 2026-08-10

**Authors:** Peng Li, Jiawei Wang, Hongyi Wang, Shuo Li, Lei Wang, Shan Gao, Chunxiao Hu, Guangjian Zhang

**Affiliations:** 1 Department of Thoracic Surgery, First Affiliated Hospital of Xi'an Jiaotong University, Yanta West Road, Xi'an, 710000, China, 86 029-85323861; 2 Department of Anesthesiology, The Affiliated Wuxi People’s Hospital of Nanjing Medical University, Wuxi, China

**Keywords:** lung transplantation, quality of life, patient-reported outcomes, PROs, symptoms

## Abstract

**Background:**

Lung transplantation (LTx) is an established treatment for patients with end-stage lung diseases and can substantially improve survival. However, posttransplant recovery involves complex physical, psychological, and functional challenges, making quality of life (QoL) an important outcome beyond survival alone. Evidence on early longitudinal QoL changes after LTx remains limited, particularly in Chinese recipients and when assessed using disease-specific patient-reported outcome instruments.

**Objective:**

This study aimed to assess QoL and track its longitudinal changes in patients who underwent LTx using a disease-specific patient-reported outcome instrument.

**Methods:**

This single-center prospective cohort study screened 66 patients for enrollment who underwent LTx at the Xi’an Jiaotong University Lung Transplantation Center in China from June 2023 to December 2025. QoL was assessed using the Chinese version of the Lung Transplant Quality of Life questionnaire at baseline (preoperative) and serially at 1, 2, 3, 4, 5, and 6 months after surgery. The Lung Transplant Quality of Life questionnaire comprises 40 items across 7 domains, and each domain score was calculated as the item mean (range 0‐4). Random-intercept linear mixed models (LMMs) with both categorical and linear time specifications were fitted separately for each domain to evaluate longitudinal changes, accounting for repeated measures and incomplete follow-up under the missing at random assumption.

**Results:**

A total of 203 interviews from 51 eligible patients were included in this study, with a median of 5 (IQR 2-6) interviews for each patient. LMM analysis revealed significant overall time effects in 6 of 7 domains (likelihood ratio test: *P*<.01 in all cases). Health perceptions showed the earliest and largest improvement (LMM-adjusted mean difference [MD] −1.58 at month 6; β=−0.23 points per month; *P*<.001), followed by respiratory symptoms (MD −0.95 at month 6; β=−0.15 per month), anxiety and depression (MD −0.82 at month 6; β=−0.13 per month), digestive symptoms (MD −0.50 at month 6; β=−0.10 per month), and cognitive limitations (MD −0.51 at month 6; β=−0.09 per month; *P*<.001 in all cases). Global well-being was the only domain with a positive trajectory (MD 1.18 at month 6; β=0.13 per month; *P*=.002), with significant improvement already evident at month 1 (MD 0.71; *P*=.23).

**Conclusions:**

LTx significantly improved QoL in patients with end-stage lung diseases, with the most substantial gains in health perceptions and respiratory symptoms. Patient-reported outcome–based monitoring can capture clinically meaningful QoL changes and should be integrated into routine posttransplant care to identify critical intervention time points and optimize long-term recovery.

## Introduction

Lung transplantation (LTx) is the definitive treatment for end-stage lung diseases, including chronic obstructive lung disease, idiopathic pulmonary fibrosis, and cystic fibrosis [1,2]. With advancements in surgical techniques and perioperative care, the 1- and 5-year survival rates have exceeded 80% and 70%, respectively, although outcomes vary across transplantation centers [1]. Despite these improvements in survival, patients after LTx continue to face multiple physical and psychological complications at various time points, including primary graft dysfunction [3], acute kidney injury [4], atrial arrhythmias [5], anastomotic fistulas [6], fungal infections [7], airway hemorrhage [8], and delirium, all of which severely undermine postoperative survival and quality of life (QoL) [9]. Current clinical practices predominantly focus on reducing postoperative complications, improving survival rates, and prolonging survival time [10,11], which may overlook the patient perspective on life quality.

This study was motivated by concerns raised by patients and their families, who frequently asked whether their symptoms and functional impairments would resolve and how these would change over time after surviving surgery. A limited body of literature has documented psychosocial and mental health outcomes after transplantation, but these studies have primarily focused on liver, heart, or kidney transplantation [12-14]. Furthermore, the assessment of QoL in patients undergoing LTx has largely relied on generic scales such as the 36-Item Short Form Health Survey or EQ-5D [15,16], which may not adequately capture the specific symptoms, functions, and lived experiences following LTx. Singer et al [17] developed the Lung Transplant Quality of Life (LT-QoL) scale in 2019, a disease-specific instrument that was subsequently translated and validated for the Chinese cultural context, thereby facilitating more accurate QoL assessment in this population.

Patient-reported outcomes (PROs) have been increasingly applied in clinical practice to monitor patient symptoms and experiences [18], improve clinical care [19], and support drug development evaluations by regulatory agencies [20]. However, very little is currently known about longitudinal QoL changes and trajectories in patients undergoing LTx based on PROs. Therefore, we conducted a prospective cohort study to assess QoL changes in patients undergoing LTx at our center using the Chinese version of the LT-QoL questionnaire and characterize the temporal patterns of recovery across multiple QoL domains.

## Methods

### Study Design and Patients

This was a single-center prospective cohort study conducted at the Xi’an Jiaotong University Lung Transplantation Center (First Affiliated Hospital of Xi’an Jiaotong University, China). A total of 66 patients who underwent LTx from June 2023 to December 2025 were screened for enrollment. Of these 66 patients, 58 (87.9%) patients or their families were fully informed about the study procedures, whereas 8 (12.1%) declined participation. Of the 58 initial patients, 7 (12.1%) were subsequently excluded (n=1 due to retransplantation and n=6 who withdrew due to being too weak to self-report), producing a final analytic cohort of 51 (87.9%) patients who contributed 203 valid interviews across 7 time points.

Inclusion criteria were (1) age of 18 years or above, (2) having undergone LTx, and (3) ability to understand the study content. Patients were excluded if they (1) underwent retransplantation or multi-organ transplantation, (2) were too weak to self-report outcomes, or (3) were diagnosed with active psychosis or severe cognitive impairment during follow-up.

### Ethical Considerations

This study was approved by the institutional review board of the First Affiliated Hospital of Xi’an Jiaotong University, China (approval number: LLSBPJ-2023-474), was exempted from clinical trial registration as it was an observational study, and was conducted in accordance with the Declaration of Helsinki. A total of 49.0% (25/51) of the patients provided written informed consent before surgery, whereas the remaining patients provided verbal informed consent due to the urgency of surgery and subsequently supplemented it with written consent at the earliest appropriate time.

### Procedures

All enrolled patients were invited to complete the LT-QoL scale at each scheduled assessment. The LT-QoL is a disease-specific questionnaire developed for the assessment of QoL in patients undergoing LTx [17]. A Chinese version was authorized and demonstrated adequate validity, reliability, and internal consistency. It consists of 40 items assessing 7 domains grouped under 4 overarching categories: symptoms (respiratory symptoms: 9 items; digestive symptoms: 8 items), health perceptions (5 items), functional impairment (sexual problems: 3 items; cognitive limitations: 4 items), and well-being (anxiety and depression: 9 items; global well-being: 2 items). Each item is quantified on a 5-point Likert scale (0‐4), with higher scores indicating greater symptom burden or impairment for all domains except global well-being, where higher scores represent better outcomes. For each domain, a composite score was calculated as the arithmetic mean of its constituent items (item mean score; range 0‐4). Further details are provided in Multimedia Appendix 1.

For each patient, assessments were scheduled before surgery (T0; baseline) and at 1, 2, 3, 4, 5, and 6 months after LTx (T1-T6). For patients who were unable to self-report, family members or caregivers completed the questionnaire based on the patient’s actual condition. Assessments were conducted in the LTx specialized outpatient clinic or via WeChat by a trained study team comprising 2 specialized nurses, 2 researchers, and 2 clinicians. Each visit could take place within a window of –7 days to +7 days from the actual scheduled appointment. Data collection was performed by a nurse under the supervision of a researcher and a clinician to ensure accuracy and completeness.

### Data Collection and Management

In this longitudinal study, data were collected and managed using the REDCap (Research Electronic Data Capture; Vanderbilt University) platform hosted at Xi’an Jiaotong University Health Science Center [21]. REDCap is a secure, web-based application designed to support data capture for research studies [22]. Patients could choose to complete either an electronic or paper-based questionnaire. Paper-based questionnaire data were entered into REDCap and verified by an independent nurse.

Demographic information and clinical characteristics were obtained from medical records supplemented by self-reported information about demographics, health behaviors, and medical history. All data were double-checked and cleaned by a professional statistician and a clinician to ensure accuracy and maintain high data quality.

### Statistical Analysis

Descriptive statistics, including means with SDs and medians with IQRs, were used to summarize patient characteristics. Continuous variables were compared using unpaired 2-tailed Student *t* tests or Wilcoxon signed-rank tests for normally and nonnormally distributed data, respectively. Categorical variables were presented as frequencies and percentages and compared using Fisher exact tests or chi-square tests as appropriate. A 2-sided *P* value of less than .05 was considered statistically significant.

To evaluate longitudinal changes in QoL scores, we used random-intercept linear mixed models (LMMs) fitted separately for each of the 7 QoL domains using restricted maximum likelihood estimation. Two complementary model specifications were used for each domain. In model 1 (categorical time), score was modeled as a function of time point + (1 | participant), where time point was treated as a categorical fixed effect with T0 (baseline) as the reference level. This model yielded the LMM-estimated marginal mean at each time point and the adjusted mean difference (MD) vs baseline with 95% CIs and Satterthwaite-approximated *P* values for each pairwise contrast. In model 2 (linear time), score was modeled as a function of month + (1 | participant), where month was treated as a continuous fixed effect (0, 1, 2,..., 6). This model estimated the average linear slope (β) per month with its 95% CI, quantifying the overall rate of change across the 6-month follow-up period.

The random-intercept specification accounted for within-patient correlation due to repeated measures and accommodated the unbalanced longitudinal design (51 patients contributing 203 interviews). All available observations were used under the missing at random (MAR) assumption, thereby mitigating potential bias from incomplete follow-up data. Individual patient trajectories overlaid with the LMM population fit are shown in Figure S1 in Multimedia Appendix 1, illustrating the heterogeneity among patients and the pattern of missing data that motivated the mixed-effects approach. The intraclass correlation coefficient (ICC; σ^2^_u_/[σu^2^_u_ + σ^2^_e_]) was calculated for each domain to quantify the proportion of the total variance attributable to between-patient differences.

The overall significance of the time effect for each domain was assessed using a likelihood ratio test (LRT) comparing the categorical time model against a null model with random intercept only (score modeled as a function of 1 + [1 | participant]). Estimated marginal means with 95% CIs were derived from model 1 for each domain at each time point. Adjusted MDs vs baseline with 95% Wald CIs were displayed as a forest plot; the linear slopes per month from model 2 were appended to the same forest plot for each domain. Individual patient trajectories were overlaid with the LMM population fit in a spaghetti plot (Figure S1 in Multimedia Appendix 1) to illustrate individual-level heterogeneity and the pattern of missing data. In addition to the domain-level analyses, item-level LMMs (score modeled as a function of time point + [1 | participant]) were fitted for all 40 individual LT-QoL items to identify specific symptoms or concerns driving the observed domain-level changes.

All analyses were performed using R (version 4.4.2; R Foundation for Statistical Computing) with the *lme4* package for LMM estimation, the *lmerTest* package for Satterthwaite df and *P* values, and the *emmeans* package for estimated marginal means and pairwise contrasts.

## Results

### Clinical Characteristics

**Figure 1.**
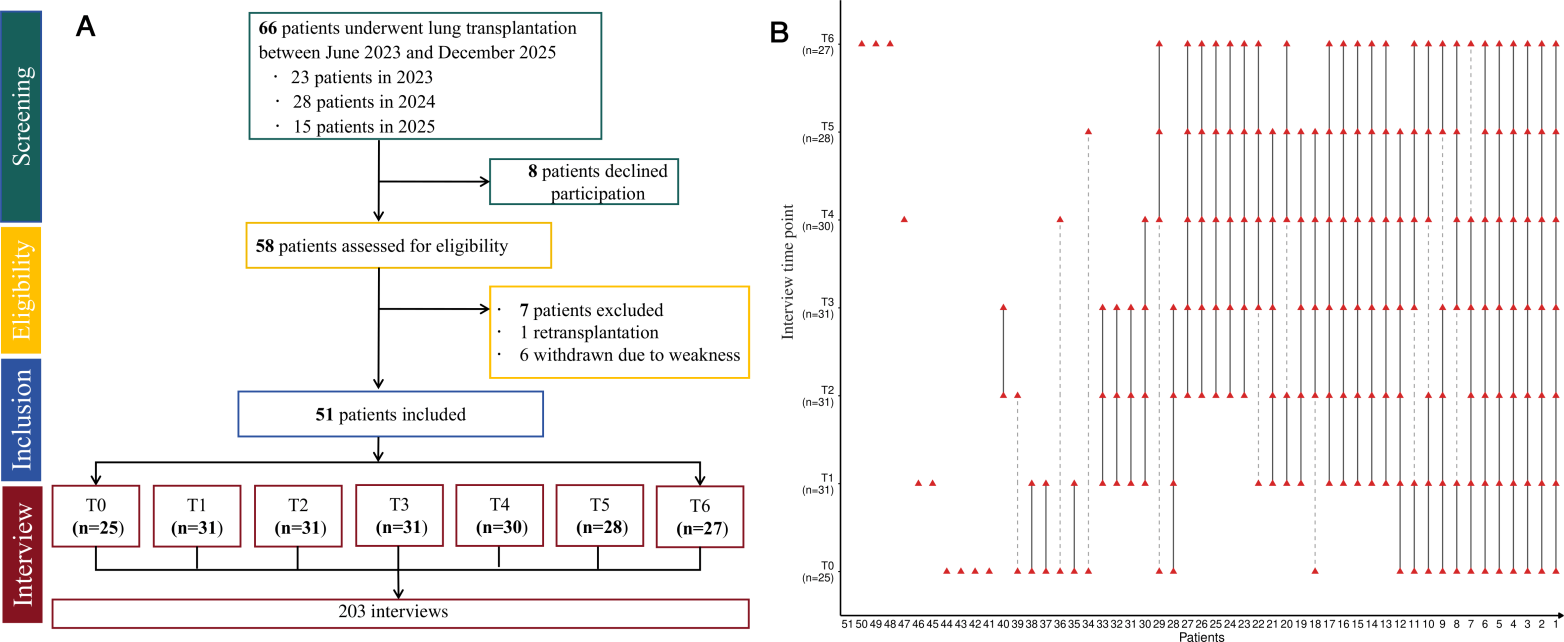
Patient enrollment and interview completion: (A) flowchart of patient screening, eligibility assessment, and inclusion and (B) interview completion for each patient across time points. Solid lines indicate consecutive follow-up visits; dashed lines indicate gaps due to missing interviews. T0: baseline (before surgery); T1 to T6: first to sixth month after lung transplantation.

Of the 66 patients who underwent LTx at our center, 15 (22.7%) were excluded: 8 (53.3%) of them declined participation, 1 (6.7%) underwent retransplantation, and 6 (40%) withdrew consent. A total of 51 eligible patients completed 203 valid QoL interviews from baseline to 6 months postoperatively. The median number of interviews per patient was 5 (IQR 2‐6), and 80.4% (41/51) and 66.7% (34/51) of the patients completed at least 2 and 3 valid interviews, respectively (Figure 1).

The mean age of the eligible patients was 56.4 (SD 9.3) years, and most were male (44/51, 86.3%) and did not have a history of smoking (39/51, 76.5%). Few of them self-reported current smoking, alcohol use, or major comorbidities (hypertension: 5/51, 9.8%; diabetes: 2/51, 3.9%; cerebrovascular or cardiovascular disease: 4/51, 7.8%). The most frequent pretransplant diagnosis was idiopathic pulmonary fibrosis (26/51, 51.0%), followed by chronic obstructive pulmonary disease (13/51, 25.5%) and pneumoconiosis (10/51, 19.6%). Mean BMI was 20.5 (SD 3.7) kg/m^2^, indicating a generally lean cohort consistent with end-stage lung disease. Bilateral sequential LTx was the most common surgery type (27/51, 52.9%), followed by single transplantation (Table 1).

**Table 1.** Demographic and clinical characteristics of the patients (N=51).

Characteristic	Values
Age (y), mean (SD)	56.4 (9.3)
Sex, n (%)	
Female	7 (13.7)
Male	44 (86.3)
BMI (kg/m^2^), mean (SD)	20.5 (3.7)
Diabetes, n (%)	2 (3.9)
CCD^a^, n (%)	4 (7.8)
Smoking history, n (%)	12 (23.5)
Hypertension, n (%)	5 (9.8)
Diagnosis, n (%)	
COPD^b^	13 (25.5)
IPF^c^	26 (51.0)
Pneumoconiosis	10 (19.6)
Others	2 (3.9)
Surgery type, n (%)	
Single LTx^d^	24 (47.1)
Bilateral LTx	27 (52.9)
ECMO^e^ type, n (%)	
VV^f^	27 (52.9)
VA^g^	12 (23.5)
VAV^h^	2 (3.9)

a CCD: cardiovascular or cerebrovascular disease.

b COPD: chronic obstructive pulmonary disease.

c IPF: idiopathic pulmonary fibrosis.

d LTx: lung transplantation.

e ECMO: extracorporeal membrane oxygenation.

f VV: veno-venous ECMO.

g VA: veno-arterial ECMO.

h VAV: veno-arterial-veno ECMO.

### Respiratory Symptoms

**Figure 2.**
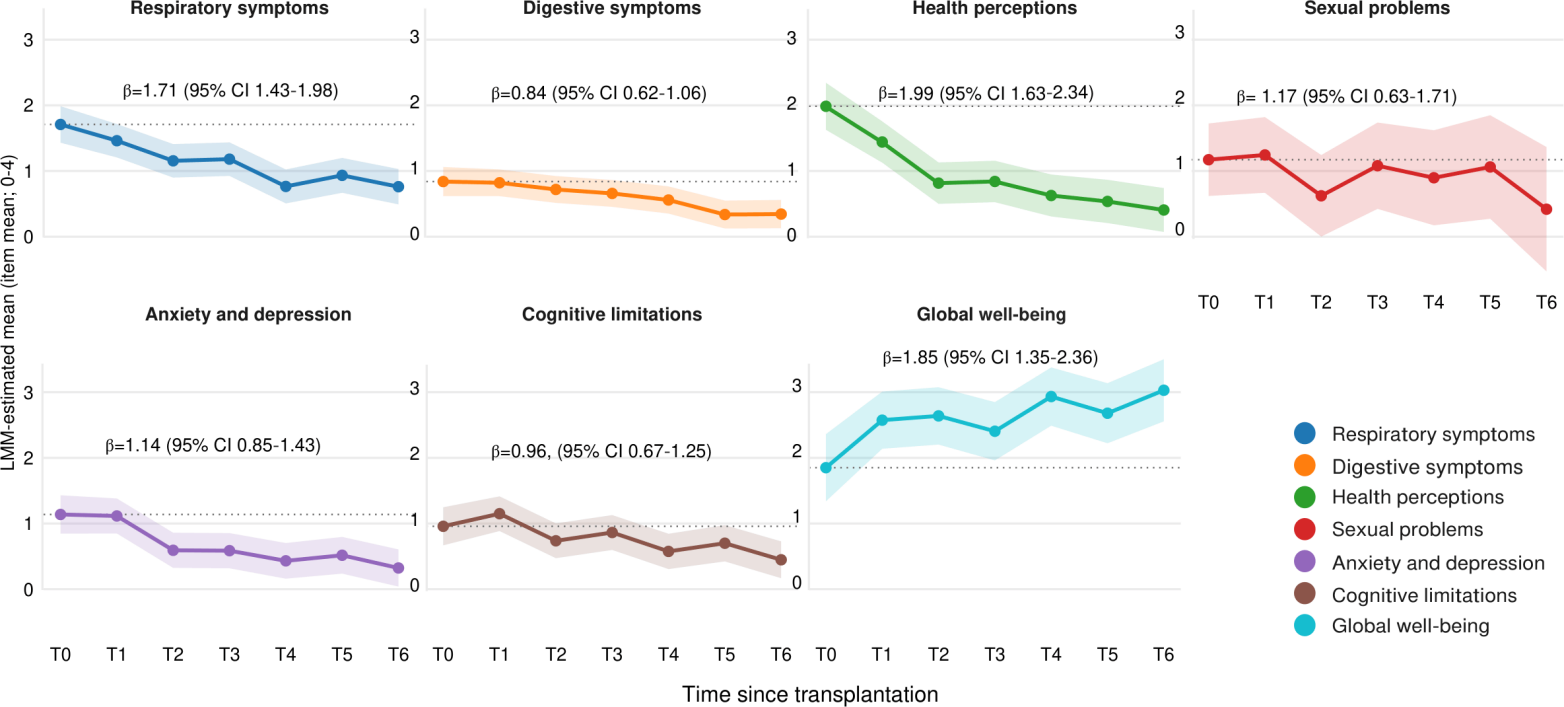
Linear mixed model (LMM)–estimated marginal means of quality of life domains over 6 months after lung transplantation. Random-intercept LMMs were fitted separately for each domain. Shaded bands represent 95% Wald CIs. Lower scores indicate better status in all domains except global well-being, for which higher scores represent better outcomes. T0: baseline (before surgery); T1 to T6: first to sixth month after lung transplantation.

In the random-intercept LMM, the model-estimated baseline mean for the respiratory symptom domain was 1.71 points (SD 0.14; 95% CI 1.43-1.98) on the item mean scale from 0 to 4 (Figure 2). The overall time effect was significant (LRT: *χ*^2^_6_=47.8; *P*<.001), and the ICC was 0.40, confirming that repeated measures within patients were substantially correlated and that the mixed-effects approach was appropriate (Table S1 in Multimedia Appendix 1).

**Figure 3.**
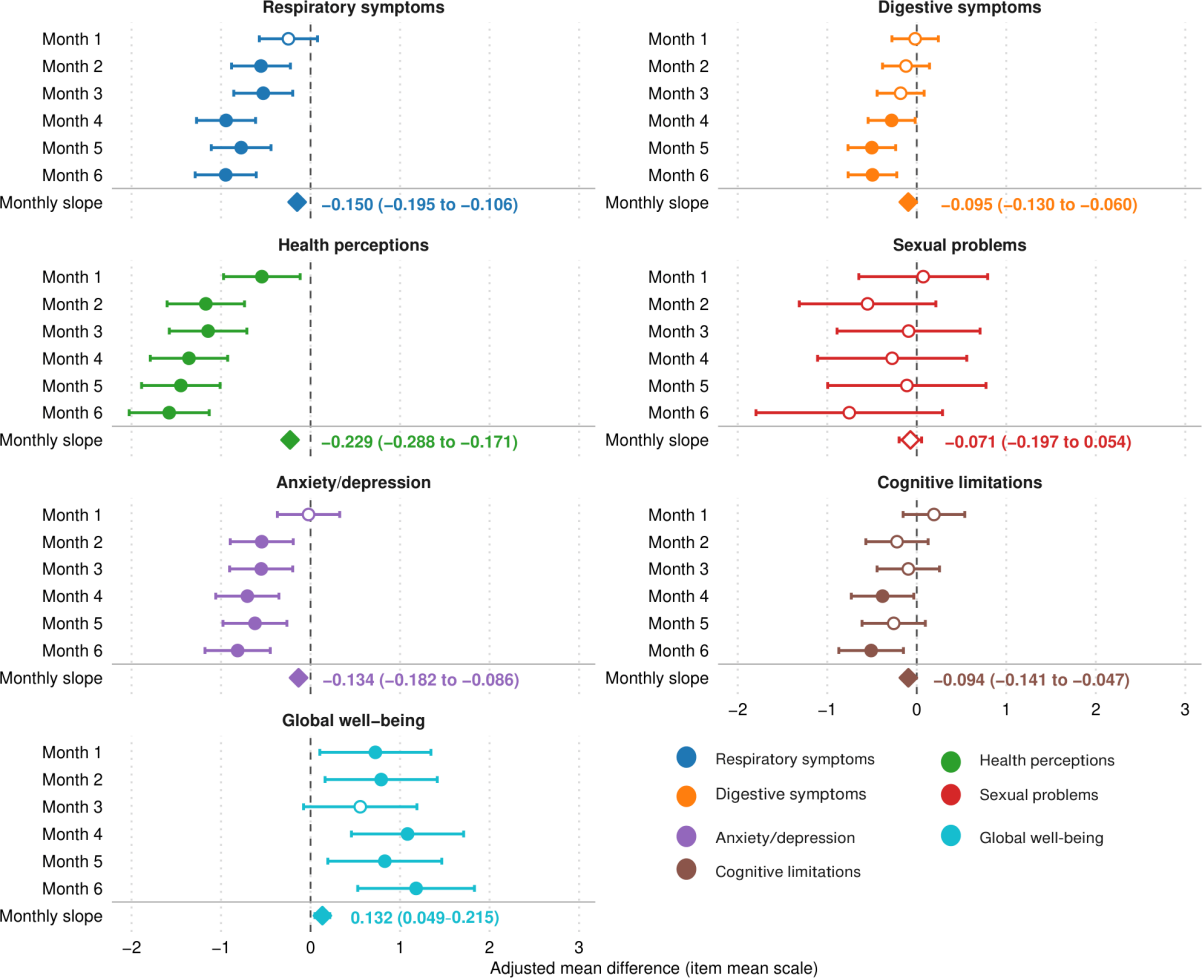
Forest plot of linear mixed model–adjusted mean differences vs baseline and linear slopes per month by quality of life domain. Circles denote time point contrasts vs baseline (model 1); diamonds denote the linear slope per month (model 2). Filled markers indicate a *P* value of .05 or less; blank markers indicate a *P* value above .05. Error bars represent 95% Wald CIs. The dashed vertical line marks the null value of 0 (no change).

The adjusted MD vs baseline was not significant at month 1 (MD −0.25, 95% CI −0.57 to 0.07; *P*=.13) but became significant from month 2 onward (MD −0.57, 95% CI −0.89 to −0.24; *P*=.001). The improvement continued to increase, reaching an MD of −0.95 (95% CI −1.29 to −0.61; *P*<.001) at month 6. The linear slope was −0.15 points per month (95% CI −0.20 to −0.11; *P*<.001), indicating a steady and clinically meaningful decline in respiratory symptoms over the 6-month follow-up period (Figure 3).

At the item level (Table S2 in Multimedia Appendix 1), the most burdensome preoperative respiratory symptoms were cough with sputum (baseline β_0_=2.79), sputum production (baseline β_0_=2.75), wheezing (baseline β_0_=2.71), and shortness of breath (baseline β_0_=2.65). These items also showed the largest LMM-adjusted 6-month improvements (shortness of breath: MD −1.65 and *P*<.001; wheezing: MD −1.58 and *P*<.001; sputum production: MD −1.22 and *P*<.001; cough: MD −1.18 and *P*<.001). Chest tightness (baseline β_0_=2.05) showed no early change (month 1: MD −0.40; *P*=.24) but a significant improvement at month 6 (MD −1.45; *P*<.001). Notably, chest pain showed a transient worsening at month 1 (MD 0.70; *P*=.02) before declining, consistent with the typical early postoperative course; however, this improvement did not reach statistical significance by month 6 (MD −0.52; *P*=.07).

### Digestive Symptoms

The estimated baseline mean for the digestive symptom domain was 0.84 points (SD 0.11; 95% CI 0.62-1.06), substantially lower than that of the respiratory symptom domain. The overall time effect was significant (LRT: *χ*^2^_6_=28.0; *P*<.001; ICC=0.41; Table S1 in Multimedia Appendix 1).

Digestive symptoms followed a slower improvement trajectory than respiratory symptoms. No significant change was detected at months 1 to 3 (*P*>.15 in all cases; Figure 2). The adjusted MD first became significant at month 4 (MD −0.28, 95% CI −0.54 to −0.02; *P*=.03) and strengthened throughout months 5 and 6 (month 5: MD −0.50, 95% CI −0.77 to −0.24, and *P*<.001; month 6: MD −0.50, 95% CI −0.77 to −0.22, and *P*<.001). The slope was −0.10 points per month (95% CI −0.13 to −0.06; *P*<.001).

At the item level (Table S2 in Multimedia Appendix 1), the most burdensome baseline digestive symptom was loss of appetite (baseline β_0_=1.66), which also showed the largest 6-month improvement (MD −1.09; *P*=.001). Fear of being far from a toilet showed both an early improvement at month 1 (MD −0.58; *P*=.005) and a sustained 6-month reduction (MD −0.77; *P*<.001). Altered taste improved significantly by month 6 (MD −0.75; *P*=.02). In contrast, stomach cramps and pain showed a transient worsening at month 1 (MD 0.45; *P*=.02) before returning to baseline levels.

### Cognitive Limitations

The estimated baseline mean for the cognitive limitation domain was 0.96 points (SD 0.15; 95% CI 0.67-1.25), with a significant overall time effect (LRT: *χ*^2^_6_=22.4; *P*=.001; ICC=0.36; Table S1 in Multimedia Appendix 1). As shown in Figures 2 and 3, the trajectory of improvement was not significant at months 1 to 3 (month 1: MD 0.19 and *P=*.17; month 3: MD −0.11 and *P*=.18). A significant reduction was first observed at month 4 (MD −0.39, 95% CI −0.73 to −0.04; *P*=.003) and persisted at month 6 (MD −0.51, 95% CI −0.87 to −0.15; *P*=.006). The slope was −0.09 points per month (95% CI −0.14 to −0.05; *P*<.001). Item-level analyses showed the largest 6-month improvements in difficulty sustaining attention (MD −0.80; *P*<.001; Table S2 in Multimedia Appendix 1) and difficulty concentrating and thinking (MD −0.50; *P*=.01), followed by slowed reactions to past events (MD −0.57; *P*=.02). General forgetfulness showed only a nonsignificant numerical decline (MD −0.25; *P*=.35).

### Sexual Problems

For the sexual problem domain, the LMM-estimated baseline mean was 1.17 points (SD 0.28; 95% CI 0.63-1.71). The overall time effect was not significant (LRT: *χ*^2^_6_=5.3; *P*=.55; Table S1 in Multimedia Appendix 1), and no individual postoperative time point reached statistical significance vs baseline (*P*>.15 in all cases; Figures 2 and 3). The slope was −0.07 points per month (95% CI −0.20 to 0.05; *P*=.27). The wide CIs reflected substantial missingness in this domain. At the item level, none of the 3 sexual problem items showed significant changes at any time point (*P*>.08 in all cases).

### Health Perceptions

The health perception domain showed the largest and earliest improvement of all 7 domains. The LMM-estimated baseline mean was 1.99 points (SD 0.18; 95% CI 1.63-2.34), the highest baseline score of any domain, indicating that concerns about health status were the most prominent source of preoperative distress. The overall time effect was highly significant (LRT: *χ*^2^_6_=65.1; *P*<.001; ICC=0.34; Table S1 in Multimedia Appendix 1).

Crucially, a significant reduction was already clear at month 1 (MD −0.55, 95% CI −0.97 to −0.12; *P*=.01), and the improvement increased monotonically thereafter, reaching an MD of −1.58 (95% CI −2.02 to −1.14; *P*<.001) at month 6 (Figures 2 and 3). The slope was the steepest among all 7 domains: −0.23 points per month (95% CI −0.29 to −0.17; *P*<.001). At the item level (Table S2 in Multimedia Appendix 1), all 5 health perception items improved significantly by month 6, such as fear of infection (MD −1.77; *P*<.001), uncertainty about future health (MD −1.76; *P*<.001), worry about worsening health (MD −1.73; *P*<.001), worry about graft failure or rejection (MD −1.68; *P*<.001), and difficulty planning for the future (MD −0.97; *P*<.001). Notably, uncertainty about future health and worry about graft rejection were already significantly lower at month 1 (MD −0.99 with *P*<.001 and MD −0.55 with *P*=.03, respectively), whereas fear of infection improved more gradually and only reached significance in month 6 (MD −1.76; *P*<.001).

### Anxiety and Depression

The estimated baseline mean for the anxiety and depression domain was 1.14 points (SD 0.15; 95% CI 0.85-1.43). The overall time effect was significant (LRT: *χ*^2^_6_=37.1; *P*<.001; ICC=0.36; Table S1 in Multimedia Appendix 1). As with respiratory symptoms, there was no detectable change at month 1 (MD −0.02; *P*=.89), but a significant improvement emerged from month 2 onward (MD −0.56, 95% CI −0.90 to −0.21; *P*=.003) and continued up to month 6 (MD −0.82, 95% CI −1.18 to −0.46; *P*<.001). The slope was −0.13 points per month (95% CI −0.18 to −0.09; *P*<.001).

Item-level analyses showed that the largest 6-month reductions were in uncontrollable worry (MD −1.27; *P*<.001), low mood (MD −1.09; *P*<.001), feeling sad (MD −0.99; *P*<.001), and irritability (MD −0.97; *P*<.001). The items reflecting more severe psychological distress, including inability to stop worrying (MD −0.64; *P*=.003), feeling guilty (MD −0.66; *P*=.001), and feeling like a burden to others (MD −0.66; *P*=.001), also improved significantly, suggesting a broad amelioration of emotional distress (Table S2 in Multimedia Appendix 1). Difficulty sleeping improved significantly by month 6 (MD −0.67; *P*=.02), although there was a nonsignificant trend toward worsening at month 1 (MD 0.51; *P*=.06). Perceived loss of interest in daily activities did not reach significance at month 6 (MD −0.41; *P*=.10).

### Global Well-Being

The global well-being domain (higher scores indicate better outcomes) was the only domain with a positive trajectory. The estimated baseline mean was 1.85 points (SD 0.26; 95% CI 1.35-2.36), and the overall time effect was significant (LRT: *χ*^2^_6_=15.7; *P*=.01; ICC=0.27; Table S1 in Multimedia Appendix 1).

The adjusted MD rose to 0.71 (95% CI 0.09-1.33; *P*=.23) at month 1, indicating that patients already perceived meaningful improvement in life quality within the first month after transplantation. The improvement peaked at month 4 (MD 1.01, 95% CI 0.46-1.71; *P*<.001) and was sustained up to month 6 (MD 1.18, 95% CI 0.53-1.83; *P*<.001). The slope was 0.13 points per month (95% CI 0.05-0.22; *P*=.002). At the item level, enjoyment of life showed a borderline improvement at month 1 (MD 0.60; *P*=.08) that became highly significant by month 6 (MD 1.27; *P*<.001). Satisfaction with current QoL improved earlier and more robustly: it was already significantly higher at month 1 (MD 0.89; *P*=.02) and continued to improve up to month 6 (MD 1.11; *P*=.004), suggesting that patients’ subjective life satisfaction recovered more rapidly than their ability to enjoy daily activities following LTx.

## Discussion

### Principal Findings

In this study, we found significant improvements in the QoL of patients undergoing LTx in the perioperative period, with the earliest and largest improvement in health perceptions followed by respiratory symptoms and anxiety and depression. In contrast, the sexual problem domain showed no significant change over time, partly attributable to high missingness and cultural reporting barriers. These findings demonstrate that LTx meaningfully improves overall QoL for patients with end-stage lung diseases across multiple domains, with clinically important reductions in symptom burden, psychological distress, and health-related concerns.

Respiratory symptoms were the predominant preoperative concern, with an estimated baseline mean of 1.71 points (95% CI 1.43-1.98), and the overall time effect was highly significant. This finding is consistent with those of previous studies linking forced vital capacity and forced expiratory volume improvements with pulmonary function recovery after transplantation [23,24]. Unlike previous studies that primarily focused on pulmonary function [25,26], we also monitored gastrointestinal symptom changes using LMMs. The digestive symptom domain showed a slower improvement trajectory than respiratory symptoms: no significant change was detected until month 4, with the improvement strengthening up to month 6. A possible explanation for this improvement may be the resolution of hypoxia and the subsequent enhancement in overall health, which collectively contribute to the restoration of normal metabolic and digestive functions [27]. Notably, stomach cramps and pain showed a transient worsening at month 1 before returning to baseline levels, and chest pain similarly worsened early. This early symptom exacerbation pattern likely reflects the combined effects of surgical stress [28], immunosuppressive medications (eg, steroids and calcineurin inhibitors) [29], and associated gastrointestinal complications. Similar gastrointestinal symptom profiles have been reported in kidney transplant recipients, with strong associations with poorer QoL [30]. Our findings support the integration of routine gastrointestinal symptom monitoring and proactive management into posttransplant care protocols.

Postoperative cognitive impairment is a common clinical phenomenon with a reported incidence of 58.3%, influenced by factors such as operation time, intensive care unit stay, pain, and cold ischemia time [31,32]. Decreases in processing speed and executive function at 6 months may signify worse survival and higher occurrence of chronic lung allograft dysfunction [33]. In contrast, the sexual problem domain was the only domain for which the LMM detected no significant overall time effect, and no individual postoperative time point reached significance vs baseline. In traditional Chinese culture, sexual health remains a sensitive and often stigmatized topic, which may lead to underreporting or social desirability bias. We did not apply analytical adjustments for potential underreporting as the degree cannot be quantified from the available data. Therefore, this null finding should be interpreted with caution. Compared to liver or kidney transplantation, where sexual problems are more prevalent [34,35], the sexual health domain in LTx remains understudied, and future research should consider culturally sensitive approaches such as anonymous electronic surveys, gender-matched interviewers, or validated proxy measures to improve assessment accuracy in Chinese transplant populations.

The health perception domain showed the earliest and largest improvement of all 7 domains, indicating that health-related concerns were the most prominent source of preoperative distress. The gradual resolution of these concerns suggests that patients experienced progressive confidence recovery. However, the persistent concern about infections despite decreasing scores underscores the challenges of living with immunosuppressive therapy. More than 30% of patients undergoing liver transplant have been reported to experience clinically significant mental distress after surgery [36], and our findings reinforce the importance of sustained psychological support throughout recovery. For the anxiety and depression domain, the adjusted improvement followed a similar delayed pattern to that of respiratory symptoms, which involved no change at month 1 but significant improvement from month 2 onward, combined with the largest item-level reductions in uncontrollable worry, low mood, and feeling sad. These findings suggest a broad amelioration of emotional distress rather than improvement in isolated symptoms.

One notable finding is the global well-being trajectory, which was the only domain with a positive slope, and the fact that the adjusted improvement was already significant at month 1. This early peak aligns with the prior longitudinal study by TenVergert et al [37], who found significant improvement in physical mobility, pain, energy, and emotional reaction at the first postoperative month. This initial peak may represent a “honeymoon effect”—an initial euphoria from surviving major surgery and receiving a new organ combined with relief from severe preoperative symptoms. However, the LMM estimates suggest a complex pattern wherein well-being peaked at month 4 rather than at month 1, and a nonsignificant dip was observed at month 3. This dip may reflect encounter with postoperative complications such as atrial arrhythmias [38], hyperglycemia [39], acute rejection [40], and medication side effects [41]. Despite this fluctuation, well-being scores remained above preoperative levels at all postoperative time points, reflecting a dynamic trajectory of initial optimism, subsequent adaptation, and long-term net improvement. Unmet life expectations after transplantation may contribute to increased stress [42]. To mitigate the emotional fluctuations observed after month 1, psychological interventions should be tailored to the early recovery phase, potentially beginning during the transition from hospital to home [43,44].

### Comparison With International Studies

The overall improvement trajectory we observed is consistent with that of the original LT-QoL validation cohort from the United States in the study by Singer et al [17], although direct numerical comparisons are limited by differences in follow-up protocols and population characteristics and our use of LMMs rather than raw score comparisons. Our findings also align with those of Ricotti et al [43], who reported significant QoL and functional improvements after LTx in a Chinese single-center study. The cultural specificities of our population, particularly regarding psychological and sexual health reporting, highlight the importance of developing culturally adapted PRO assessment strategies for diverse transplant populations.

### Clinical Implications

Our findings carry several important clinical implications. First, the LMM analysis identified month 1 as a critical window characterized by transient worsening in chest pain and stomach cramps, whereas global well-being simultaneously peaked. This paradoxical pattern characterized by symptom worsening alongside perceived improvement suggests that targeted gastrointestinal symptom management and immunosuppressive protocol adjustment should be implemented during this period without assuming that patients’ optimism reflects symptom resolution. Second, the delayed cognitive improvement (significant only from month 4) highlights the need for continued monitoring and rehabilitation throughout the recovery period. Long-term tracking of cognitive function changes is important for capturing early signals of impairment, allowing for timely implementation of targeted interventions such as cognitive training, rehabilitation, and support group therapy [45]. Postoperative rehabilitation, including physical therapy, aerobic exercise, and resistance training, can also help patients adapt to their new life [46]. Third, our findings demonstrate that PRO-based monitoring using disease-specific instruments and LMM analysis can capture clinically meaningful QoL changes that may not be detectable by standard clinical assessments alone. The integration of routine PRO monitoring into posttransplant care can facilitate early identification of patients requiring additional support, enable personalized care planning, and improve shared decision-making between clinicians and patients. In our clinical practice, we have established a peer support program named the LTx Patients Association, which invites well-recovered transplant recipients to provide guidance and emotional support to recent transplant recipients, encouraging social reintegration through community-based activities.

### Limitations

Several limitations should be acknowledged. First, our data were derived from a relatively small cohort at a single center with follow-up limited to 6 months. Although we used LMM, which appropriately handles incomplete data under the MAR assumption, survivorship bias remains a concern in that patients who remained in follow-up may represent a healthier subset, potentially leading to overestimation of QoL improvement at later time points. Second, the reasons for missing data are multifactorial, including patient death, geographic barriers to follow-up, health deterioration preventing participation, and the urgency of transplant surgeries precluding baseline assessment for some patients. While our LMM approach provides valid inference under the MAR assumption and the ICCs (0.25‐0.41 across domains) confirmed the appropriateness of the mixed-effects framework, unmeasured confounding related to differential attrition cannot be ruled out. Third, while the LT-QoL questionnaire has been validated for the Chinese context, certain domains, particularly sexual function, may be subject to cultural response bias, as discussed above. The high missingness in this domain severely limits statistical power. Additionally, the relatively short follow-up period may not capture long-term QoL fluctuations. Future studies should implement strategies to minimize attrition, establish multicenter cohorts, extend follow-up duration, and develop culturally tailored assessment approaches to comprehensively evaluate QoL in Chinese patients undergoing LTx.

### Conclusions

In this prospective cohort study, LTx significantly improved overall QoL in patients with end-stage lung diseases over 6 months, as demonstrated by random-intercept LMM analysis across 7 QoL domains. The health perception domain showed the earliest and largest improvement, followed by respiratory symptoms, anxiety and depression, and cognitive limitations. Additionally, the first postoperative month represents a critical intervention window for gastrointestinal symptom management and psychological support. These findings support the integration of disease-specific PRO monitoring and LMM-based longitudinal analysis into posttransplant clinical care to identify critical intervention time points, personalize supportive care, and optimize long-term recovery outcomes.

## Supplementary material

10.2196/86211 Multimedia Appendix 1 Supplementary figures, tables, and details of the lung transplantation management procedure.

## Abbreviations

ICC: intraclass correlation coefficient
LMM: linear mixed model
LRT: likelihood ratio test
LT-QoL: Lung Transplant Quality of Life
LTx: lung transplantation
MAR: missing at random
MD: mean difference
PRO: patient-reported outcome
QoL: quality of life
REDCap: Research Electronic Data Capture
