# Supplementary material for: Patient-Reported Quality of Life in Lung Transplantation: Prospective Cohort Study
Source: Interact J Med Res. 2026 Aug 10;15:e86211. doi: 10.2196/86211 (PMC13457444; doi:10.2196/86211)
Supplement: Supplementary file 1 [file ijmr-v15-e86211-s001.docx]

**Supplementary Materials**

***Quality of Life in Lung Transplantation***

Health-related quality of life (HRQOL) is an individual’s or a group’s perceived physical and mental health over time. It is usually collected by standardized questionnaires directly from patients. Furthermore, LTx patients may report vastly different HRQL due to heterogeneity of health conditions. Lung transplant Quality of Life (LT-QOL) is published in 2019 as a questionnaire exclusively designed for LTx patients. The LT-QOL is a new, multidimensional instrument that characterizes and quantifies HRQOL. It contains 10 scales that measure symptoms, health perceptions, functioning, and well-being. We leveraged it to attain more sensitive specific and personalized results. In consideration of the cultural disparities and language impediments, the Chinese version of the questionnaire was employed to circumvent potential misunderstandings and ensure accurate conveyance of participants’ responses. The multidimensional LT-QOL is a novel instrument specific for lung transplantation, with excellent evidence of content and construct validity and internal consistency[1]. Consisting of 40 items, the Chinese version has been officially authorized by LT-QOL developers. The content validity index of the total scale is 0.912, and the content validity index of each item is between 0.781 and 1.0. The Cronbach's α coefficient for total LT-QOL score was 0.947, and 0.710~0.929 for every common factor. It is thoroughly verified and possess adequate validity, retest reliability and internal consistency.[2] Each item asked respondents about the frequency of occurrence of symptoms (e.g., 1 = never to 5 = very often) or the extent to which a statement applied (e.g., 1 = not at all to 5 = very much) by the 5-level Likert scale.

**Reference**

1.	Singer JP, Soong A, Chen J et al. Development and Preliminary Validation of the Lung Transplant Quality of Life (LT-QOL) Survey. *American Journal of Respiratory and Critical Care Medicine* 2018; 199: 1008.

2.	LIANG Yongchun, ZHOU Haiqin, ZHU Xuefen, CAO Xiaodong, MIN Qunhui, HUANG Keyao. Validation of Chinese version of the Lung Transplant Quality of Life survey for lung transplant recipients. *Chinese Journal of Nursing* 2022; 57: 688.

***Clinical care protocol***

All participants received a standardized immunosuppression regimen at our institution. Basiliximab (20mg) was administered intraoperatively for induction of immunosuppression. Methylprednisolone was used intraoperatively and during the first 3 days after transplant, with the dose adjusted according to the type of transplant procedure (single lung transplantation: 500mg per day; double lung transplantation: 750mg per day). Thereafter, prednisone was used instead starting from the 4th day after transplantation, at a dose equivalent to that of methylprednisolone. Then, the dose was gradually reduced by 5mg per week until a maintenance dose of 10-15mg was reached after transplant. In the event of acute rejection, methylprednisolone (10mg/kg per day) was used for the first 3 days, and prednisone (60mg) was started on the 4th day and gradually tapered to the maintenance dose.

Tacrolimus was started on post-operative day (POD) 1 and continued indefinitely after transplant, with the dose adjusted according to the trough level. The targeted tacrolimus trough levels decreased gradually, being 10-15mg/L and 8-12mg/L at 6 months and 12 months after transplant respectively, and maintained at 6-10mg/L thereafter. The monitoring frequency was as follows: once a week from POD 1 to 3 months after transplant; once every 2 weeks from 3 to 6 months after transplant; and once a month more than 6 months after transplant.

Mycophenolate mofetil was started on the day of transplant, at a dose of 250-750mg twice daily, and continued indefinitely.

Sirolimus was added in select cases of recurrent acute rejection or chronic lung allograft dysfunction (CLAD) at the discretion of the treating clinicians, or, in rare cases, replaced tacrolimus when patients developed chronic kidney disease.

**Table S1.** Random-intercept linear mixed model variance components, intraclass correlation coefficients, and likelihood-ratio tests for the overall time effect, by quality-of-life domain.

| **QoL domain** | *σ*^2^_u_ | *σ*^2^_e_ | **ICC** | *χ*^2^ (LRT) | *df* | *P* value |
| --- | --- | --- | --- | --- | --- | --- |
| Respiratory symptoms | 0.225 | 0.338 | 0.40 | 47.35 | 6 | < 0.001 |
| Digestive symptoms | 0.147 | 0.214 | 0.41 | 27.59 | 6 | < 0.001 |
| Health perceptions | 0.282 | 0.558 | 0.34 | 64.09 | 6 | < 0.001 |
| Sexual problems | 0.623 | 1.176 | 0.34 | 5.19 | 6 | 0.519 |
| Anxiety/depression | 0.218 | 0.391 | 0.36 | 36.08 | 6 | < 0.001 |
| Cognitive limitations | 0.217 | 0.382 | 0.36 | 22.20 | 6 | 0.001 |
| Global well-being | 0.387 | 1.179 | 0.25 | 16.69 | 6 | 0.011 |

**Abbreviations:** ICC, intraclass correlation coefficient; LRT, likelihood-ratio test; QoL, quality of life.

*σ*^²^_u_, between-patient (random intercept) variance; *σ*^²^_e_, within-patient (residual) variance. ICC = σ²u / (σ²u + σ²e). The LRT compares the categorical-time model (score ~ timepoint + (1|subject)) against a null model (score ~ 1 + (1|subject)).

| **Table S2.** Item-level LMM results: baseline estimates and adjusted mean differences at month 1 and month 6 versus baseline. | | | | | | | |
| --- | --- | --- | --- | --- | --- | --- | --- |
| **Domain / Item** | **Item** | **Description** | **β_0_** | **T1 Δ** | **T1 *P*** | **T6 Δ** | **T6 *P*** |
| **Respiratory symptoms** | LQ1 | Shortness of breath | 2.65 | −0.96 | **0.006** | −1.66 | **< 0.001** |
|  | LQ2 | Wheezing | 2.71 | −0.89 | **0.012** | −1.58 | **< 0.001** |
|  | LQ3 | Chest tightness | 2.05 | −0.40 | 0.243 | −1.45 | **< 0.001** |
|  | LQ4 | Chest pain | 0.70 | 0.62 | **0.024** | −0.52 | 0.070 |
|  | LQ5 | Cough | 2.79 | −0.46 | 0.145 | −1.18 | **< 0.001** |
|  | LQ6 | Sputum production | 2.75 | −0.52 | 0.118 | −1.22 | **< 0.001** |
|  | LQ7 | Difficulty swallowing food | 0.47 | 0.18 | 0.425 | −0.16 | 0.489 |
|  | LQ8 | Difficulty swallowing liquids | 0.20 | 0.25 | 0.188 | −0.06 | 0.778 |
|  | LQ9 | Choking/coughing when swallowing | 1.04 | −0.28 | 0.191 | −0.72 | **0.002** |
| **Digestive symptoms** | LQ10 | Taste disturbance | 1.13 | −0.03 | 0.920 | −0.75 | **0.020** |
|  | LQ11 | Loss of appetite | 1.66 | −0.14 | 0.655 | −1.09 | **0.001** |
|  | LQ12 | Acid reflux | 0.52 | −0.21 | 0.264 | −0.26 | 0.194 |
|  | LQ13 | Nausea/vomiting | 0.76 | −0.27 | 0.229 | −0.38 | 0.100 |
|  | LQ14 | Abdominal bloating | 0.95 | 0.36 | 0.190 | −0.52 | 0.071 |
|  | LQ15 | Stomach cramps/pain | 0.28 | 0.45 | **0.022** | −0.14 | 0.486 |
|  | LQ16 | Fear of being far from toilet | 1.01 | −0.58 | **0.005** | −0.77 | **< 0.001** |
|  | LQ17 | Diarrhea | 0.44 | 0.17 | 0.461 | −0.04 | 0.863 |
| **Health perceptions** | LQ18 | Worry about worsening health | 2.19 | −0.61 | **0.022** | −1.73 | **< 0.001** |
|  | LQ19 | Uncertainty about future health | 2.17 | −0.99 | **< 0.001** | −1.76 | **< 0.001** |
|  | LQ20 | Fear of infection | 2.36 | −0.46 | 0.159 | −1.77 | **< 0.001** |
|  | LQ21 | Worry about graft failure/rejection | 2.05 | −0.55 | **0.030** | −1.68 | **< 0.001** |
|  | LQ22 | Difficulty planning for the future | 1.16 | −0.16 | 0.491 | −0.97 | **< 0.001** |
| **Sexual problems** | LQ23 | Unable to relax and enjoy sex | 1.18 | 0.03 | 0.930 | −0.66 | 0.235 |
|  | LQ24 | Loss of sexual desire | 1.30 | −0.03 | 0.937 | −0.94 | 0.087 |
|  | LQ25 | Difficulty becoming sexually aroused | 1.04 | 0.20 | 0.579 | −0.67 | 0.205 |
| **Anxiety/depression** | LQ26 | Feeling sad | 1.40 | −0.20 | 0.333 | −0.99 | **< 0.001** |
|  | LQ27 | Low mood | 1.47 | −0.21 | 0.364 | −1.09 | **< 0.001** |
|  | LQ28 | Uncontrollable worry | 1.42 | −0.34 | 0.116 | −1.27 | **< 0.001** |
|  | LQ29 | Sadness affecting daily life | 0.78 | 0.07 | 0.749 | −0.64 | **0.003** |
|  | LQ30 | Feeling tense/anxious | 0.84 | 0.44 | 0.064 | −0.41 | 0.099 |
|  | LQ31 | Feeling drained/exhausted | 1.09 | 0.51 | 0.057 | −0.67 | **0.015** |
|  | LQ32 | Feeling fearful | 0.84 | −0.11 | 0.573 | −0.66 | **0.001** |
|  | LQ33 | Difficulty relaxing | 0.83 | 0.06 | 0.739 | −0.66 | **0.001** |
|  | LQ34 | Irritability | 1.55 | −0.51 | 0.064 | −0.97 | **< 0.001** |
| **Cognitive limitations** | LQ35 | Forgetfulness | 1.18 | 0.06 | 0.822 | −0.25 | 0.348 |
|  | LQ36 | Slowed reactions | 0.96 | 0.17 | 0.454 | −0.57 | **0.018** |
|  | LQ37 | Difficulty sustaining attention | 0.99 | 0.17 | 0.428 | −0.80 | **< 0.001** |
|  | LQ38 | Difficulty concentrating/thinking | 0.72 | 0.32 | 0.095 | −0.50 | **0.014** |
| **Global well-being†** | LQ39 | Able to enjoy life | 1.86 | 0.59 | 0.090 | 1.26 | **< 0.001** |
|  | LQ40 | Satisfied with current QoL | 1.80 | 0.87 | **0.016** | 1.09 | **0.004** |

**Figure S1. Individual trajectories with LMM population fit** (Grey lines: individual patients (N = 51); colored line with shading: LMM marginal mean 95% CI.)

西安交通大学第一附属医院胸外科

肺移植队列

LTx cohort in NWC

(Lung Transplantation cohort in the NorthWest of China)

调查量表（随访）

| 姓名： |  |
| --- | --- |
| 编号： | LTx |
| 住院号/门诊号： |  |
| 移植日期： | 年 月 日 |
| 填表日期： | 年 月 日 |

随访记录说明

复查（随访）计划安排表，手术日期	**年	月	日**

| **V1（ 年 月 日）术后1周** | | | | **V2（ 年 月 日）术后2周** | | | | **V3（ 年 月 日）术后3周** | | | |
| --- | --- | --- | --- | --- | --- | --- | --- | --- | --- | --- | --- |
| 检验 | 检查 | 样本 | 问卷 | 检验 | 检查 | 样本 | 问卷 | 检验 | 检查 | 样本 | 问卷 |
|  |  |  | × |  |  |  | × |  |  |  | × |
| **V4（ 年 月 日）术后1月** | | | | **V5（ 年 月 日）术后2月** | | | | **V6（ 年 月 日）术后3月** | | | |
| 检验 | 检查 | 样本 | 问卷 | 检验 | 检查 | 样本 | 问卷 | 检验 | 检查 | 样本 | 问卷 |
|  |  |  |  |  |  |  |  |  |  |  |  |
| **V7（ 年 月 日）术后4月** | | | | **V8（ 年 月 日）术后5月** | | | | **V9（ 年 月 日）术后6月** | | | |
| 检验 | 检查 | 样本 | 问卷 | 检验 | 检查 | 样本 | 问卷 | 检验 | 检查 | 样本 | 问卷 |
|  |  | × |  |  |  | × |  |  |  |  |  |
| **V10（ 年 月 日）术后7月** | | | | **V11（ 年 月 日）术后8月** | | | | **V12（ 年 月 日）术后9月** | | | |
| 检验 | 检查 | 样本 | 问卷 | 检验 | 检查 | 样本 | 问卷 | 检验 | 检查 | 样本 | 问卷 |
|  |  | × | × |  |  | × | × |  |  | × |  |
| **V13（ 年 月 日）术后10月** | | | | **V14（ 年 月 日）术后11月** | | | | **V15（ 年 月 日）术后12月** | | | |
| 检验 | 检查 | 样本 | 问卷 | 检验 | 检查 | 样本 | 问卷 | 检验 | 检查 | 样本 | 问卷 |
|  |  | × | × |  |  | × | × |  |  |  |  |
| **V16（ 年 月 日）术后15月** | | | | **V17（ 年 月 日）术后18月** | | | | **V18（ 年 月 日）术后21月** | | | |
| 检验 | 检查 | 样本 | 问卷 | 检验 | 检查 | 样本 | 问卷 | 检验 | 检查 | 样本 | 问卷 |
|  |  | × |  |  |  | × |  |  |  | × |  |
| **V19（ 年 月 日）术后24月** | | | | **V20（ 年 月 日）术后30月** | | | | **V21（ 年 月 日）术后36月** | | | |
| 检验 | 检查 | 样本 | 问卷 | 检验 | 检查 | 样本 | 问卷 | 检验 | 检查 | 样本 | 问卷 |
|  |  |  |  |  |  | × |  |  |  |  |  |
| 备注： | | | | | | | | | | | |

# 复查/随访

随访时间：	年	月	日，记录人：

患者自行预约挂号复诊，需要保持空腹状态直至要求空腹的检查或样本采集血液、尿液）完成，在	时完成的步骤包括：

1.调查问卷：

2.临床检验：

3.临床检查：

4.生物样本采集：血液□、尿液□、粪便□、痰液□

请复查患者自主填写生活质量和睡眠质量调查，行动不便的患者请家属或护士询问填写。**生活质量和睡眠质量是患者术后康复评估的重要参考依据之一，对于患者之后治疗方案的调整至关重要，任何人不得代为填写！**填写完之后请交到门诊，请妥善保管，不得丢失。

**1.肺移植术后生活质量评估（术后：	）**

我们想要了解您在过去1个月内，是否出现以下症状或情况。请您选择最贴近真实的每一个条目，谢谢。

- 没有出现：表示一点也没有或从来没有出现。
- 偶尔出现：表示有一点或一小部分时间出现，但不是非常罕见。
- 适中出现：表示一个月有几次出现
- 经常出现：表示一星期内有几次出现或一般频繁
- 每次出现：表示几乎每次都出现或非常频繁。

| **症状** | **没有**  出现 | **偶尔**  出现 | **适中**  出现 | **经常**  出现 | **每次**  出现 |
| --- | --- | --- | --- | --- | --- |
| 1. 我有呼吸急促 |  |  |  |  |  |
| 2. 我有气喘发作 |  |  |  |  |  |
| 3. 我感到胸闷 |  |  |  |  |  |
| 4. 我感到胸痛 |  |  |  |  |  |
| 5. 我平时有咳嗽 |  |  |  |  |  |
| 6. 我有痰液咳出 |  |  |  |  |  |
| 7. 我吞咽食物有困难 |  |  |  |  |  |
| 8. 我吞咽液体有困难 |  |  |  |  |  |
| 9. 我吞咽时有呛咳/咳嗽 |  |  |  |  |  |
| 10. 我对味觉的改变感到困扰 |  |  |  |  |  |
| 11. 我胃口不好/没有食欲 |  |  |  |  |  |
| 12. 我有反酸 |  |  |  |  |  |
| 13. 我有恶心呕吐 |  |  |  |  |  |
| 14. 我有胃部胀气 |  |  |  |  |  |
| 15. 我有胃部痉挛或疼痛 |  |  |  |  |  |
| 16. 我总是害怕离厕所太远 |  |  |  |  |  |
| 17. 我有腹泻 |  |  |  |  |  |

我们想要了解您在过去1个月内的健康认识和功能方面的情况。请您选择最贴近真实的每一个条目，谢谢。

- 没有出现：表示一点也没有或从来没有出现。
- 偶尔出现：表示有一点或一小部分时间出现，但不是非常罕见。
- 适中出现：表示一个月有几次出现
- 经常出现：表示一星期内有几次出现或一般频繁
- 每次出现：表示几乎每次都出现或非常频繁。

| **健康认识/功能** | **没有**  出现 | **偶尔**  出现 | **适中**  出现 | **经常**  出现 | **每次**  出现 |
| --- | --- | --- | --- | --- | --- |
| 18. 我担心我的健康会变差 |  |  |  |  |  |
| 19. 我对自己未来的健康感到不确定 |  |  |  |  |  |
| 20. 我担心发生感染 |  |  |  |  |  |
| 21. 我担心我的肺移植无效或者发生排斥 |  |  |  |  |  |
| 22. 肺移植手术后，我对计划未来有困难 |  |  |  |  |  |
| 23. 无法放松和享受性生活 |  |  |  |  |  |
| 24. 性欲缺乏 |  |  |  |  |  |
| 25. 难以唤起性兴奋 |  |  |  |  |  |

我们想要了解您在过去1个月内的情绪和情感方面的情况。请您选择最贴近真实的每一个条目，谢谢。

- 没有出现：表示一点也没有或从来没有出现。
- 偶尔出现：表示有一点或一小部分时间出现，但不是非常罕见。
- 适中出现：表示一个月有几次出现
- 经常出现：表示一星期内有几次出现或一般频繁
- 每次出现：表示几乎每次都出现或非常频繁。

| **情绪和情感体验** | **没有**  出现 | **偶尔**  出现 | **适中**  出现 | **经常**  出现 | **每次**  出现 |
| --- | --- | --- | --- | --- | --- |
| 26. 我感到沮丧 |  |  |  |  |  |
| 27. 我情绪低落 |  |  |  |  |  |
| 28. 我不能停止或控制住担心 |  |  |  |  |  |
| 29. 沮丧的感觉影响到我的日常生活 |  |  |  |  |  |
| 30. 我感觉紧张、焦虑或不安 |  |  |  |  |  |
| 31. 我感到无精打采，感觉身心被掏空 |  |  |  |  |  |
| 32. 我感到害怕，好像有什么可怕的事情要发生 |  |  |  |  |  |
| 33. 我很难放松自己 |  |  |  |  |  |
| 34. 我容易生气或易激惹 |  |  |  |  |  |
| 35. 我容易健忘，如忘记最近发生的享情，忘记东西放哪了 |  |  |  |  |  |
| 36. 我对说过和做过的事情反应迟钝 |  |  |  |  |  |
| 37. 我在任何活动上长时间集中注意力有困难 |  |  |  |  |  |
| 38. 我做集中注意力和思考的事有困难 |  |  |  |  |  |
| 39. 我能够享受生活 |  |  |  |  |  |
| 40. 我满意我现在的生活质量 |  |  |  |  |  |

2. **睡眠质量（术后：	）**

患者您好，肺移植术后患者常因不良并发症引起不同程度的睡眠问题，请您如实填写您的睡眠质量情况，以便全面评估您的病情。请注意：所有项为必填项，感谢您的配合，祝您早日康复。

**睡眠质量：**1=很好 2=较好 3=较差 4=很差

**您晚上一般___点睡觉，入睡需___分钟，早晨一般___点起床，**

**一夜实际睡眠时间__小时**

**近一个月，您有没有因下列情况而影响睡眠 （a~j条均问，如果没有填“0”）**

a入睡难（30分钟内未入睡）（__次/周）b疼痛不适（__次/周）c夜间去厕所（__次/周）d呼吸不畅/困难（__次/周）e大声咳嗽或鼾声高（__次/周）f感觉冷（__次/周）g感觉热（__次/周）h做噩梦（__次/周）i夜间易醒或早醒（__次/周）

j其他______（__次/周）

**近1个月您是否经常使用催眠药物才能入睡？**

1=无 2=l次/周 3=2~3次/周 4=≥4次/周

**近l个月您是否常感到身体困倦？**

1=无 2=l次/周 3=2~3次/周 4=≥4次/周

**近1个月您做事的是否心累、力不从心？**

1=没有 2=偶尔有 3=有时有 4=经常有
